# Spectrum-Effect Relationships between Fingerprints of *Caulophyllum robustum* Maxim and Inhabited Pro-Inflammation Cytokine Effects

**DOI:** 10.3390/molecules22111826

**Published:** 2017-10-26

**Authors:** Shaowa Lü, Shuyu Dong, Dan Xu, Jixin Duan, Guoyu Li, Yuyan Guo, Haixue Kuang, Qiuhong Wang

**Affiliations:** 1 Key Laboratory of Chinese Materia Medica, Heilongjiang University of Chinese Medicine, Ministry of Education, Harbin 150040, China; lswa5599@hotmail.com (S.L.); shuyuzuibang@163.com (S.D.); xu_dan199205@163.com (D.X.); duan_jixin@yeah.net (J.D.); guoyuyan622@163.com (Y.G.); 2 Pharmaceutical College, Harbin University of Commerce, Harbin 150086, China; Leegy@163.com; 3 Pharmaceutical College, Guangdong Pharmaceutical University, Guangzhou 510224, China

**Keywords:** *Caulophyllum robustum* Maxim, spectrum-effect relationship, collagen-induced arthritis (CIA) model, multiple linear regression (MLR), gray relational analysis (GRA)

## Abstract

*Caulophyllum robustum* Maxim (CRM) is a Chinese folk medicine with significant effect on treatment of rheumatoid arthritis (RA). This study was designed to explore the spectrum-effect relationships between high-performance liquid chromatography (HPLC) fingerprints and the anti-inflammatory effects of CRM. Seventeen common peaks were detected by fingerprint similarity evaluation software. Among them, 15 peaks were identified by Liquid Chromatography-Mass Spectrometry (LC-MS). Pharmacodynamics experiments were conducted in collagen-induced arthritis (CIA) mice to obtain the anti-inflammatory effects of different batches of CRM with four pro-inflammation cytokines (TNF-α, IL-β, IL-6, and IL-17) as indicators. These cytokines were suppressed at different levels according to the different batches of CRM treatment. The spectrum-effect relationships between chemical fingerprints and the pro-inflammation effects of CRM were established by multiple linear regression (MLR) and gray relational analysis (GRA). The spectrum-effect relationships revealed that the alkaloids (*N*-methylcytisine, magnoflorine), saponins (leiyemudanoside C, leiyemudanoside D, leiyemudanoside G, leiyemudanoside B, cauloside H, leonticin D, cauloside G, cauloside D, cauloside B, cauloside C, and cauloside A), sapogenins (oleanolic acid), β-sitosterols, and unknown compounds (X3, X17) together showed anti-inflammatory efficacy. The results also showed that the correlation between saponins and inflammatory factors was significantly closer than that of alkaloids, and saponins linked with less sugar may have higher inhibition effect on pro-inflammatory cytokines in CIA mice. This work provided a general model of the combination of HPLC and anti-inflammatory effects to study the spectrum-effect relationships of CRM, which can be used to discover the active substance and to control the quality of this treatment.

## 1. Introduction

Rheumatoid arthritis (RA) is a chronic, systemic inflammatory disease that results in swollen joints, pain, and the disorder of many tissues and organs [1]. RA may affect other parts of the body and even cause a low red blood cell count, as well as inflammation around the lungs and heart [2]. Although the mechanism of RA is not completely understood, laboratory and clinical evidence suggests that genetic factors and pro-inflammatory cytokines play important roles in its pathogenesis [3]. Currently, there are no ideal methods or medicines to heal it, but anti-inflammatories and analgesics are effective ways to relieve RA symptoms. Traditional Chinese Medicine (TCM) has achieved a large number of results to varying extent in the treatment of RA [4]. To illustrate the pathology of RA, many models have been made to mimic the human RA process, among which the collagen-induced arthritis (CIA) model is the most widely used [5,6]. Unlike other experimental arthritis models, the CIA model resembles human RA more closely in terms of the clinical, histological, and immunological features, as well as in terms of the genetic linkage [7].

*Caulophyllum robustum* Maxim (CRM), also known as “Hong Mao Qi”, is an extremely effective Chinese folk medicine to treat muscle pain, rheumatoid arthritis, menoxenia, menstrual abdominal pain, postpartum bleeding pain, tonsillitis, etc. Its roots and rhizomes are the main medicinal parts [8]. *Caulophyllum thalictroides*, a related plant in the same genus, is also widely used to relieve pain, induce childbirth, and rectify delayed or irregular menstruation in America [9]. Further, *C. thalictroides* extract and its main components can suppress the levels of COX-2, iNOS, TNF-α, IL-1β, and IL-6 [10,11]. Similarly, our previous work found that *C. robustum* extract had a noticeable anti-inflammatory effect in adjuvant arthritis rats and suppressed the level of IL-1β, IL-4, IL-10, TNF-α, IL-17, and IFN-γ, which may be one of its principal anti-RA mechanisms. Moreover, CRM extract can significantly counteract the allergy induced by 2,4-dinitrofluorobenzene (DNFB), reduce the percentages of CD3^+^ and CD4^+^, and increase CD8^+^ [12]. Forty-five compounds have been isolated from CRM, which are mainly made up of triterpenoid saponins and alkaloids [3,13]. However, due to its complicated active components, it is sometimes difficult to define the anti-RA active components and their contribution rate of synergistic effect. Therefore, it is of great necessity to make clear what the effective compositions are during clinical application.

In recent years, the spectrum-effect relationship has been established as a new method that can combine HPLC with pharmacological effects to rapidly target functional constituents [14]. Spectrum-effect relationships can explain the correlation between the fingerprint and the efficacy of TCM, as well as provide a platform for the determination of a material basis for pharmacological function. It has been proposed and successfully applied to the screening and analysis of multiple bio-active compounds in herbal medicines [15].

There are a variety of statistical methods employed to establish a spectrum-effect relationship, including correlation analysis (CA), principal component analysis (PCA), canonical correlation analysis (CCA), multiple linear regression (MLR), gray relational analysis (GRA), partial least squares (PLS), and so on [16]. Among them, GRA can judge the size of the relevance between the efficacy index and the chromatographic peak, and offers a possibility for predicting the active components [17]. When discussing the contribution rate of each ingredient, MLR can more clearly point out the combined effect of TCM on the efficacy index, quantitatively describe the relationship between the two, and provide evidence for the basic research of pharmacodynamics [18]. Therefore, the rational use of data processing and analysis methods will play a positive role in the spectrum-effect relationship study of Chinese medicine.

To further elucidate the material basis of the anti-inflammatory effect of CRM, this paper established the HPLC fingerprint and compared the anti-inflammatory activity of different batches of CRM through pharmacological studies of CIA mice. At the same time, the correlation between the common peaks and the anti-inflammatory activity was calculated by MLR and GRA. According to the correlation coefficient, the main pharmacological components were clarified and the contribution of each component to anti-inflammatory activity was determined, which provided reference for CRM quality control and new drug research.

## 2. Results and Discussion

### 2.1. Results of the HPLC Experiment

#### 2.1.1. HPLC Experiments

To optimize the HPLC method, we investigated the important influencing factors such as different absorption wavelengths, different mobile phases, time, and column temperature. The results showed that more peaks with obvious characteristics appeared at 206 nm compared with 220 nm, 254 nm, and 306 nm. Moreover, using 0.1% aqueous phosphoric acid solution as the mobile phase, the fingerprint was better than that achieved using distilled water. At the same time, all peaks were found before 50 min. Furthermore, the column temperature had a great influence on the spectrum, and the results showed that the peak width and resolution of each sample met the requirements at 30 °C.

**Figure 1:**
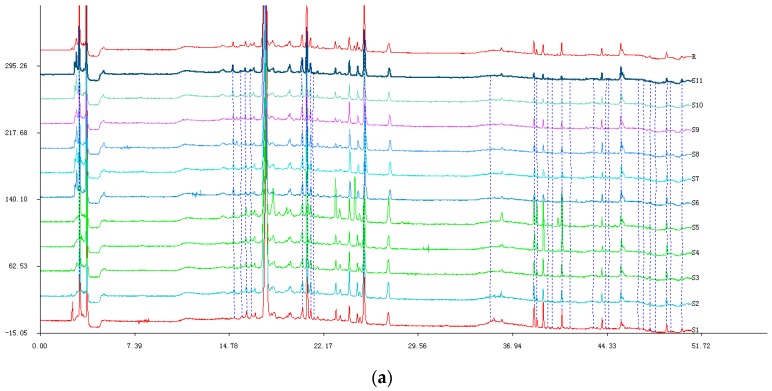

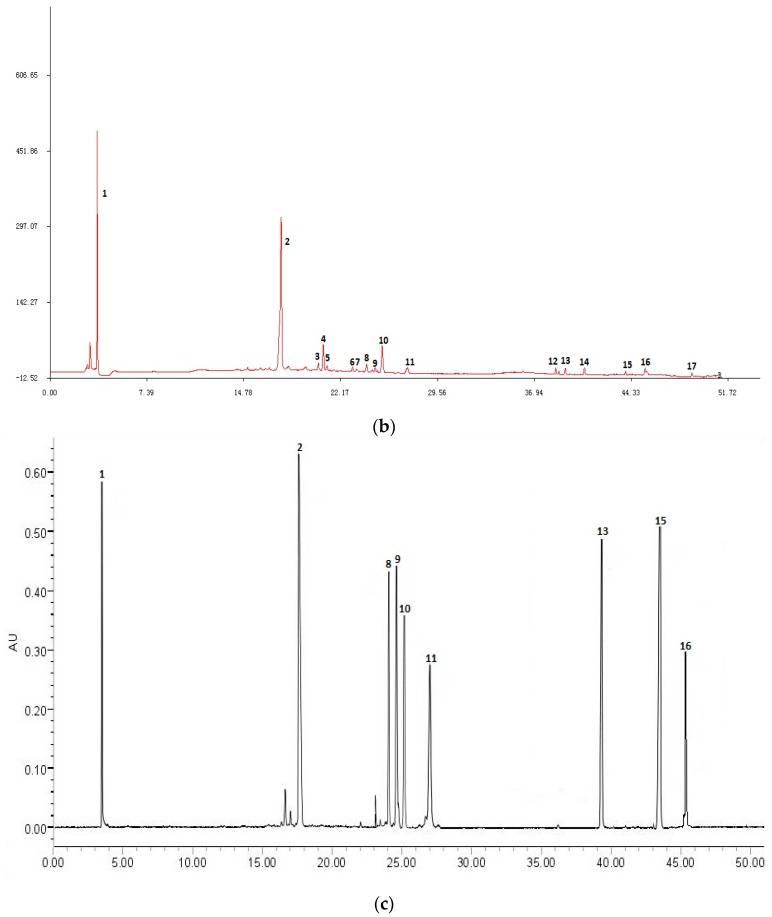
(**a**) HPLC fingerprint; (**b**) the mutual mode; and (**c**) mixed reference substances.

The results of the methodology validation showed that the relative retention time (t_R_) of the precision was in the range of 0.03–0.24% and 1.15–2.82% for peak areas. For the repeatability experiment, it was in the range of 0.02–0.20% for t_R_ and 1.56–2.50% for peak areas. For the stability experiment, it was below 2.27% for t_R_ and 2.03% for peak areas. These results indicated that the method used for the HPLC fingerprint was valid and suitable. There were 17 common peaks found by comparing the ultraviolet spectra and HPLC retention time from the samples (Figure 1a). HPLC of mixed reference substances and the fingerprints of different batches of CRM were obtained under the optimized condition as shown in Figure 1b,c. Cauloside D (X11), whose retention time varied more slightly than others, was selected as the reference peak to calculate the relative retention time and the relative peak area of the other 16 common peaks among different batches of CRM (Table 1 and Table 2). When compared with the reference substances, there were nine characteristic peaks identified. They were X1: *N*-methylcytisine (t_R_ = 3.671), X2: Magnoflorine (t_R_ = 17.720), X8: Cauloside H (t_R_ = 24.181), X9: Leonticin D (t_R_ = 24.804), X10: Cauloside G (t_R_ = 25.328), X11: Cauloside D (t_R_ = 27.220), X13: Cauloside C (t_R_ = 39.347), X15: Oleanic acid (t_R_ = 43.940), and X16: β-sitosterols (t_R_ = 45.422). The other peaks which could not be identified were preliminarily supposed to be peak 3 as the composition of alkaloids, and peaks 4, 5, 6, 7, 12, 14, and 17 as the composition of saponins, according to the ultraviolet spectrum.

The values of the relative standard deviation (RSD%) of the relative retention time of the common characteristic peaks were all less than 1.00%, but the values of the RSD% of the relative peak area of the common characteristic peaks were in the range of 18.14–59.66%, showing that the content of each sample varied significantly from different production areas and different harvest times. A large number of experimental studies demonstrated that the effect of Chinese medicine would be influenced by its growth environment [19]. The 11 batches of CRM were harvested at three different locations, each at a different altitude, including Sichuan Province (32°52’), Hebei Province (38°24’), and Heilongjiang Province (46°63’) in China. As the sunshine per day and growth periods changed, the contents of CRM effective constituents were different. Moreover, the amount of rain also had a great influence on the medicinal materials in CRM; for example, the precipitation amount was greater in Heilongjiang Province in 2014 than in 2013. Therefore, this may be a reason why the common peak areas and the contents of effective components in the 11 batches of CRM were different.

#### 2.1.2. Similarity of Fingerprints

The similarity between the fingerprints of different batches of CRM and the reference fingerprints were: 0.977, 0.935, 0.985, 0.945, 0.984, 0.965, 0.979, 0.987, 0.985, 0.976, and 0.971, respectively (Table 3), which matched closely with the requirements of the similarity. However, the similarity between **S2** and **S4**, **S6**, and **S11** was 0.862, 0.891, and 0.891, which indicated that the content of the chemical components was different between **S2** and **S4**, **S6**, and **S11**, respectively. Combined with the fingerprint, it can be seen that the differences between the common fingerprint peak levels and types among **S4**, **S11**, and **S2** fingerprints were small, but **S6** and **S2** showed a larger difference. Therefore, it can be concluded that collection time and area had a strong influence on the chemical composition and content of CRM.

#### 2.1.3. Results of Hierarchical Clustering Analysis (HCA)

**Figure 2:**
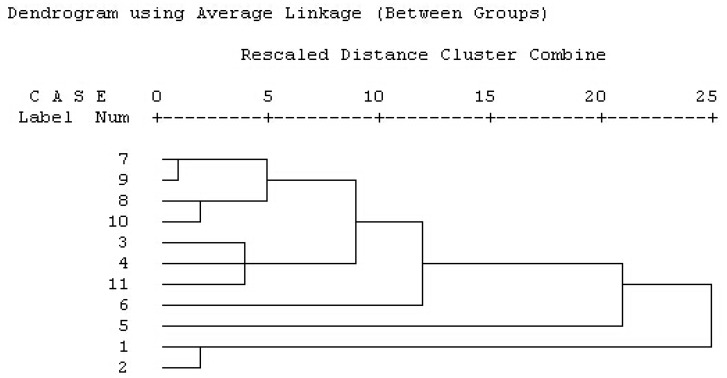
The results of HCA.

From the dendrogram (Figure 2), it can be easily seen that **S1** and **S2**, collected from Dazhou Mountain in Sichuan Province, China, are clustered in I, which revealed that their properties were homogenous. At the same time, **S7**, **S9**, **S8**, and **S10**, collected from Suiling Zhangjiawan forest farm in Heilongjiang Province, China, and **S3**, **S4**, **S11**, collected from Mao County in Sichuan and Suiling Zhangjiawan forest farm in Heilongjiang, were similar to **S5** and **S6**, coming from Anguo City in Hebei Province China, and thus were all clustered in II. Therefore, we can conclude that some critical factors, such as collecting time and area, could play the same important role in influencing the quality of CRM.

### 2.2. The Results of CIA Pharmacodynamics Experiment

#### 2.2.1. Effect of CRM on Gross Lesions of CIA Mice

**Figure 3:**
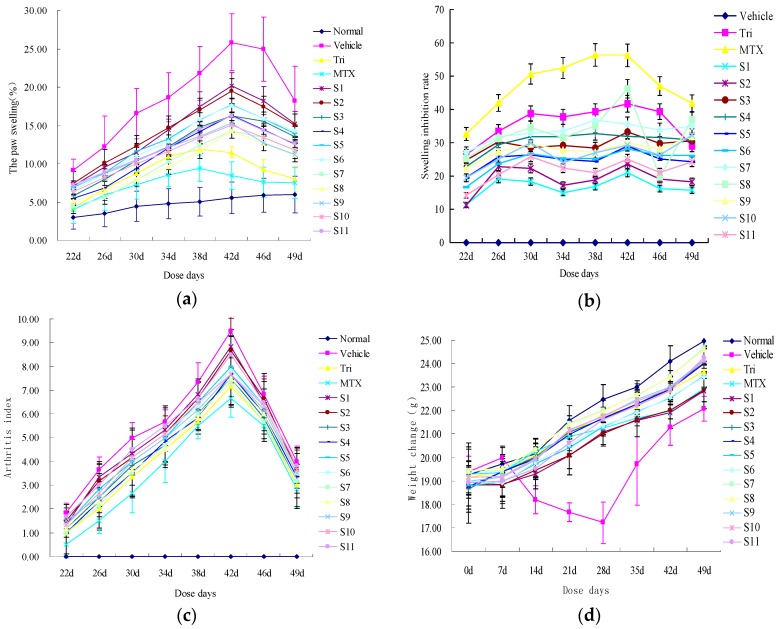
Anti-inflammatory activity index of (**a**) paw swelling (%); (**b**) swelling inhibition rate; (**c**) arthritis index (AI)’ and (**d**) body weight change.

The anti-inflammation effect of each of the samples was tested by the hind paw swelling rate (Figure 3a,b), arthritis index(AI) (Figure 3c), and weight change (Figure 3d). Methotrexate (MTX) tablets are often used as a treatment medicine for some autoimmune diseases such as RA [20], although they are usually associated with side effects. Tri tablets, a kind of Chinese medicinal plant isolated from *Tripterygium wilfordii* Hook f., is very effective for RA treatment [21]. As MTX and Tri tablets are commonly used in clinical applications, we used them as positive drugs to evaluate CRM. Compared with the MTX group and the Tri group, all the different batches of CRM (**S1**–**S11**)-medicated groups could reduce the hind paw swelling rate and AI, and better protect against weight loss in CIA mice. The samples originating from different areas showed different anti-inflammation and inhibition effects. For example, **S1**–**S4**—collected from Sichuan Provinces—showed significantly lower effects than **S7**–**S11**, collected from Heilongjiang Province (*p* ≤ 0.05). With the altitude of the harvest place increasing, the peak areas of **S7**–**S11** also increased, showing better inhibitory effects. As shown in Figure 3a,b, **S5** exhibited the strongest inhibitory effects, as this sample was collected in an abundant rainfall year (2014) in Heilongjiang Province. Moreover, **S9** and **S10** showed similar inhibition effects because they shared the same harvest time and location. According to the results, it can be inferred that different harvest times and collection areas resulted in different levels of anti-inflammation activity.

#### 2.2.2. Effect of CRM on Sera Cytokines of CIA Mice

**Figure 4:**
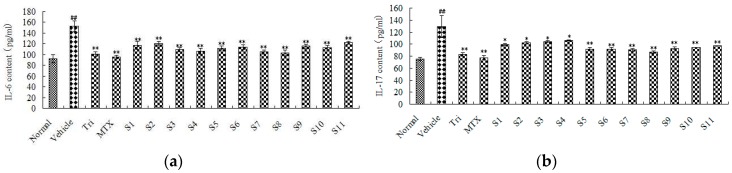

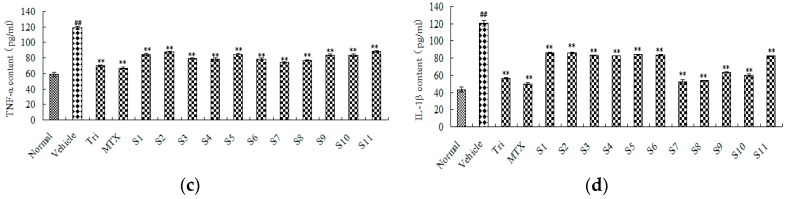
The levels of pro-inflammatory cytokines: (**a**) TNF-α content; (**b**) IL-1β content; (**c**) IL-6 content; and (**d**) IL-17 content. ^##^: *p* < 0.01 was significant difference between vehicle group and the control group; *: *p* < 0.05 was considered statistically significant between vehicle group and treatment groups; ** : *p* < 0.01 was significant difference between vehicle group and treatment groups.

ELISA was used to evaluate the anti-inflammatory activity by analyzing the content change of TNF-α, IL-1β, IL-6, and IL-17 in mice sera. These pro-inflammatory cytokines were involved in the whole process of inflammation. The levels of TNF-α, IL-1β, IL-6, and IL-17 were found to be apparently increased in the sera of the vehicle group compared to the normal group (*p* < 0.01). Similar to the MTX and Tri groups, these pro-inflammatory cytokines were also suppressed at different levels in CIA mice treated with all of the different batches of CRM (Figure 4a–d). It can be seen that different collecting times and areas resulted in different CRM anti-inflammatory effects, and the difference in the inhibition of the expression of TNF-α and IL-1β was more obvious in the multiple comparison Student-Newman-Keuls (SNK) test results (Table 4, Table 5, Table 6 and Table 7). The analysis results were as follows: (1) With TNF-α content used as an index, the anti-inflammatory effect between **S7** and **S8** had the same efficacy and no obvious difference (*p* = 1.000); **S4**, **S6**, and **S3** had the same efficacy and no obvious difference (*p* = 0.107); **S10**, **S9**, **S5**, and **S1** had the same efficacy and no obvious difference (*p* = 0.783); **S2** and **S11** had the same efficacy and no obvious difference (*p* = 0.510); **S7** and **S8** had a greater effect with significant difference (*p* < 0.05); (2) With IL-1β content used as an index, **S11**, **S4**, **S3**, **S6**, and **S5** had the same efficacy and no obvious difference (*p* = 0.395); **S5**, **S1**, and **S2** had the same efficacy and no obvious difference (*p* = 0.072); **S7**, **S8**, **S9**, and **S10** exhibited a greater effect with significant difference (*p* < 0.05), and **S7** was the most effective; (3) With IL-6 content used as an index, **S8**, **S7**, **S4**, **S3**, **S5**, and **S10** were more effective with no obvious difference (*p* = 0.055); **S7**, **S4**, **S3**, **S5**, **S10**, and **S6** had the same efficacy and no obvious difference (*p* = 0.071); **S4**, **S3**, **S5**, **S10**, **S6**, and **S9** had the same efficacy and no obvious difference (*p* = 0.060); **S3**, **S5**, **S10**, **S6**, **S9**, and **S1** had the same efficacy and no obvious difference (*p* = 0.148); **S5**, **S10**, **S6**, **S9**, and **S2** had no obvious difference (*p* = 0.139); **S6**, **S9**, **S1**, **S2**, and **S11** had the same efficacy and no obvious difference (*p* = 0.085); (4) With IL-17 content used as an index, **S7**, **S8**, **S5**, **S6**, **S9**, and **S10** were more effective with no obvious difference (*p* = 0.165); **S7**, **S5**, **S6**, **S9**, **S10**, **S11**, and **S1** had the same efficacy and no obvious difference (*p* = 0.084); **S10**, **S11**, **S1**, and **S2** had the same efficacy and no obvious difference (*p* = 0.059); **S11**, **S1**, **S2**, **S3**, and **S4** had the same efficacy and no obvious difference (*p* = 0.092). At the same time, the results of the combined efficacy of the different batches of CRM showed that the effects of CRM from Heilongjiang Province were the best under the same collection time, and the effects of CRM were collected in 2014 were the best under the same collection origin.

In exploring Chinese medicinal plants in the treatment of RA, pro-inflammation cytokines are likely to be targets for the inhibition of RA. There are many types of pro-inflammation cytokines. For instance, TNF-α is an autocrine stimulator as well as a potent paracrine inducer of pro-inflammatory mediators; it can induce nitric oxide (NO) production and release prostaglandin E2 (PGE2) by synovial cells [22]. Similar to TNF-α, IL-1 increases the production of matrix metalloproteinases (MMPs) by chondrocytes which, in turn, causes joint damage in RA patients [23]. IL-6 shows multiple effects of inflammatory cytokines, which are produced by T cells, monocytes, macrophages, and synovial fibroblasts. IL-17 promotes neutrophil differentiation, maturation, activation, and cytokine release, mononuclear cells activation and cytokine release, the production of prostaglandins, and MMPS synthesis. It plays a role in the inflammation process because it can produce TNF-α, IL-1β, IL-6, and so on [24,25]. To sum up, TNF-α, IL-1β, IL-6, and IL-17 can induce related cytokines in the form of network formation, having an impact on RA.

### 2.3. The Results of the Spectrum-Effect Relationships

#### 2.3.1. Stepwise Multiple Linear Regression

STEPWISE regression equations were established by analyzing the independent variable of peaks areas (X) and the dependent variable of sera cytokine content (Y). For instance, there was a linear interrelationship between X2, X6, and Y_TNF-α_; the determination coefficient was 0.879, and the residuals follow a normal distribution. The results of the variance analysis showed statistical significance (*p* < 0.05).
$Y_{\mathrm{TNF}-\alpha }=81.274+4.144E^{-6}X2-1.322E^{-6}X6$R^2^ = 0.879*p* < 0.05$Y_{\mathrm{IL}-1\beta }=142.699-5.132E^{-5}X1-8.390E^{-5}X3-2.529E^{-5}X6$R^2^ = 0.983$Y_{\mathrm{IL}-6}=177.487-2.927E^{-5}X1-7.309E^{-5}X13$R^2^ = 0.941$Y_{\mathrm{IL}-17}=102.588-1.941E^{-5}X1+4.744E^{-5}X10$R^2^ = 0.755

#### 2.3.2. Partial Correlation Analysis

**Figure 5:**
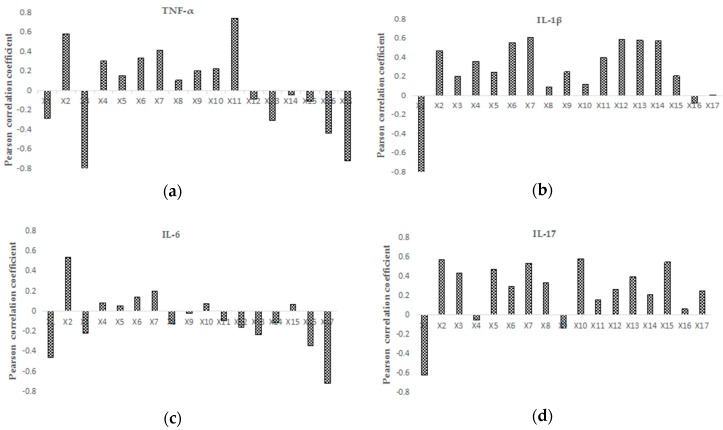
Pearson correlation coefficients between the peak area and inflammatory markers, (**a**) TNF-α; (**b**) IL-1β; (**c**) IL-6; and (**d**) IL-17.

The relationships between the peak area and inflammatory markers were reflected by the Pearson correlation coefficient (Figure 5). We analyzed the peaks whose correlation coefficients were greater than 0.5, and the results showed that the correlations between the peaks and cytokine content were considerably different. For instance, there was a positive correlation between X2, X11, and TNF-α, and there was a negative correlation between X3 and X17.

#### 2.3.3. Integration of the Analytical Results

The union of the STEPWISE multiple linear regression and partial correlation analysis indicated that the common peaks of different pharmacodynamic indicators were also different (Table 8). This indicated that the anti-inflammatory effects came from the cooperative action of CRM’s multiple components.

#### 2.3.4. Gray Relational Analysis

Furthermore, GRA can examine the correlation of each peak and anti-inflammatory effect directly. The results showed that the correlation from high to low was: X17 > X15 > X12 > X14 > X13 > X9 > X7 > X16 > X6 > X5 > X11 > X8 > X3 > X4 > X10 > X1 > X2 (Table 9). There was a definite corresponding relation between the chemical composition of each component in the HPLC fingerprint and the given component’s anti-inflammatory effects. This also showed that the anti-inflammatory effects came from the cooperative action of CRM’s multiple components. The known components identified in this study that had higher connectivity with anti-inflammatory effect (>0.71) include X15 (oleanic acid), X12 (cauloside B), X14 (cauloside A), and X13 (cauloside C).

### 2.4. Assignments of the Correlated Peaks

Peak identification and assignment in HPLC fingerprints were made on the basis of the reference substances in the chromatogram, and a total of nine characteristic peaks were identified, shown in Figure 1 and Table 10 [26,27].

*C. robustum* had an anti-RA effect on CIA mice, which was confirmed in our previous research (in publication). Seventeen alkaloids had been isolated and identified, such as *N*-methylcytisine, magnoflorine, taspine, hongmaoxinjian, epimediphine, and (+)-reticuline. Twenty-eight saponins had been isolated and identified from CRM, and the main saponins included calouside A–H [7,28]. Caulophyllum triterpenes had been detected at very high levels, amounting to up to 7.46% of the total dry weight in root and rhizome [29].

On the one hand, cauloside A–D could significantly inhibit the expression of pro-inflammatory factors, for instance, TNF-α, IL-1β, and IL-6 [30,31,32]. Additionally, cauloside C can improve the level of the release of amino acids so as to increase the penetrability of the yeast cell serous membrane [33,34]. Cauloside C can also increase the leakage on the plasma membrane permeability, of which it has the highest ultraviolet absorption at 260 nm from the cells [35,36].

Previous studies showed that saponins in CRM had four main kinds of aglycone, such as caulophyllogenin, hederagenin, oleanolic acid, and echinocystic acid [26]. Oleanolic acid produced a marked reduction in complementary levels and inflammatory effects on carrageenan-induced paw edema in rats when injected intraperitoneally [37]. Moreover, the oleanolic acid type of pentacyclic triterpenoids always had anti-inflammation activities. For example, platycodin D and D3 could inhabit early inflammation by the regulation of medium PEG2 expression in the model brought about by tissue plasminogen activator (TPA) [38,39]. Hederagenin also had an anti-inflammatory effect and dose-dependently reduced the lipopolysaccharide-induced mRNA levels of iNOS and COX-2, as well as of NO, PGE2, TNF-α, IL-1β, and IL-6 cytokines [40].

On the other hand, the anti-inflammation effects of alkaloids from *C. robustum* have also been reported. One hundred micromoles of magnoflorine show NO-suppressing activity and can inhibit the expression of IL-6 in lipopolysaccharides (LPS)-treated RAW264.7 cells [41]. *N*-metylcytisine can inhibit carrageenan-induced paw edema in rats, comparable to the reference drug diclofenac, and its anti-inflammatory properties of the most active amines are due to accommodation in the COX-1/COX-2 active sites [42].

In addition, β-sitosterol can relieve inflammation by raising the levels of the NO gastric mucosa protection factor and inhibiting the release and accumulation of TNF-α [43]. The results mentioned in the above reports were consistent with the results in the present study.

## 3. Materials and Methods

### 3.1. Materials and Reagents

CRM samples were mostly purchased from the Chinese herbal medicine markets in several provinces of China (Table 11), and their species were identified by Professor Zhenyue Wang (College of Pharmacy, Heilongjiang University of Chinese Medicine, Harbin, China). Reference standards of *N*-methylcytisine, magnoflorine, and oleanolic acid were obtained from the National Institute for the Control of Pharmaceutical and Biological Products of China (Beijing, China). We prepared cauloside H, leonticin D, cauloside G, cauloside D, cauloside C, and β-sitosterol in our laboratory, and all of their purities were greater than 98%.

Acetonitrile and methanol of HPLC grade were purchased from Dikma (Beijing, China). Glacialaceticacid, phosphoricacid, and triethylamine of analytic reagent (AR) grade were bought from Tianjin Kemiou Chemical Reagent Co., Ltd. (Tianjin, China). Deionized water was obtained from a Milli-Q water purification system (Millipore China Limited, Shanghai, China).

Bovine type II collagen and complete Freund's adjuvant (CFA) were purchased from Sigma (St. Louis, MO, USA). TNF-α, IL-1β, IL-6, and IL-17 were purchased from R and D Systems in USA. MTX tablets were purchased from SPH Sine Pharmaceutical Laboratories Co., Ltd. (Shanghai, China), lot number: 03614040, standard: 2.5 mg. Tri tablets were purchased from Grand Pharmaceutical Huangshi Feiyun Pharmaceutical Co., Ltd., (Huangshi, Hubei, China) lot number: 20131001, standard: 10 mg. 

Male DBA/1 mice (6–8 weeks old, weighing 18–22 g) were purchased from HFK BIOSCIENCE Co., Ltd. (Beijing, China). Temperature, humidity, and light conditions in the rat environment were kept constant, with food and water provided ad libitum. All mice were acclimated in the laboratory for at least one week prior to the experiment. Before testing, animals were fasted overnight with free drinking water. All animal experiments were carried out in accordance with the Guidelines for the Care and Use of Laboratory Animals, and were approved by the Animal Ethics Committee of Heilongjiang University of Chinese Medicine.

### 3.2. Sample Preparation

The dry roots and rhizomes of CRM were soaked for 12 h with 10 times 80% ethanol, and then extracted three times, each time for about 1.5 h. The filter liquor was combined and reclaimed by a rotary evaporator (Büchi R-210, Buchi Laboratory Equipment Trading Ltd. Shanghai, China), followed by freeze-drying. The power was dissolved into acetonitrile/water (50:50, *v*/*v*), and filtered through a 0.22-μm membrane to give a sample solution at a concentration of 0.5 mg/mL for use with HPLC.

Mixed standard solutions containing *N*-methylcytisine, magnoflorine, cauloside H, Leonticin D, cauloside G, cauloside D, cauloside C, oleanolic acid, and β-sitosterol were prepared by adding an accurately weighed amount of each standard stock into a volumetric flask, which was dissolved into 10 mL acetonitrile/water (50:50, *v*/*v*), and then filtered through a 0.22-μm membrane to yield mixed standard solutions.

### 3.3. Analysis of HPLC Fingerprints

#### 3.3.1. HPLC Conditions

The samples were injected into a 2695 HPLC system (Waters, Milford, MA, USA) with a 2996 photodiode array detector (PAD). The chromatographic separation was performed using a Venusil XBP C18(L) column (250 mm × 4.6 mm id, 5 μm, Agela Technologies, Beijing, China), operated at 30 °C. With the mobile phase comprising acetonitrile (A) and 0.1% aqueous phosphoric acid solution (B), gradient elution was developed under the following conditions: 0–6 min, 5% A; 6–20 min, 5–30% A; 20–30 min, the mobile phase composition maintained at 30% A; 30–45 min, 30–100% A; and 45–50 min, the mobile phase composition maintained at 100% A. The flow rate was controlled at 1.0 mL/min. The detection wavelength was set at 206 nm, with a sample injection volume of 20.0 μL.

#### 3.3.2. Validation of Methodology

To achieve a reproducible and stable chemical fingerprint of CRM, the precision, repeatability, and stability of the HPLC method was evaluated. Using the established HPLC condition programs, the precision was analyzed by six successive injections of sample solutions and the repeatability of six different samples was assessed by the best peak shapes, responses, and peak resolution in preliminary experiments, respectively. In addition, the analysis of different time periods in a day (0, 2, 4, 8, 12, 24 h) was used to evaluate the stability of the same test solution over 24 h. The relative standard deviation (RSD) of the relative retention time and relative peak area of the characteristic peaks were calculated to evaluate the method, respectively.

#### 3.3.3. Similarity of HPLC Analysis

Taking different batches of CRM extract, according to Section 3.3.1., we detected and recorded the chromatogram and its characteristic data. The fingerprint similarity of CRM was evaluated by the Chinese traditional medicine fingerprint similarity calculation software, which was recommended by the Chinese Pharmacopoeia Committee (Version 2004A) using the median method.

#### 3.3.4. HCA of CRM Fingerprints

The HCA is a multivariate statistical analysis method for classification. The basic idea is as follows: first, take each sample as a category, select the most similar samples as a class, then choose the similarly larger sample and classify, and continue for all samples [44]. The ultimate goal is to maximize the similarity between the comparable data elements and to maximize the difference between different types of data elements. In this part, the HCA of different batches of CRM was performed using SPSS statistical analysis software (SPSS for Windows 16.0, SPSS, Palo Alto, CA, USA) based on the between-groups linkage method and squared Euclidean distance [45].

### 3.4. Anti-Inflammatory Effect Experiment

#### 3.4.1. The Establishment of CIA Model, Compound Administration, and Grouping

Ninety DBA/1 mice were divided into 15 groups (n = 6 for each group). One of the groups served as “normal”, whereas the other 14 groups were subjected to CIA induction. Bovine type II collagen was dissolved in 0.1 M acetic acid to prepare 2 mg/mL mixed liquor, which was maintained overnight at 4 °C. This mixture and an equal volume of CFA were emulsified at a low temperature. The DBA/1 mice were immunized intradermally 1–2 cm from the base of the tail with 100 μL hybrid emulsion (containing 100 μg of bovine type II collagen), which was treated as the first immunity on day 0. Then, mice were boosted at the base of the tail, avoiding the primary immune location, with 100 μg bovine types II collagen that was emulsified in an equal volume of CFA on day 21, which was treated as the second immunity. Tri tablets, MTX tablets, and CRM extracts (**S1**–**S11**) were dissolved in distilled water and were oral administered once per day for 30 days from day 21 until day 51. According to the amount of human dosage and the conversion relationship between human and mouse, the dose of Tri tablets and MTX tablets was 11.375 mg/kg and 0.975 mg/kg, respectively. The dose was 100.0 mg/kg of different batches from CRM on the basis of some preliminary experiments on dose selection. The groupings were named as follows: normal group (normal), CIA model group (vehicle), Tri group, MTX group, and different batches of CRM group.

#### 3.4.2. Morphological Analysis

Morphological parameters such as weight, arthritic index/score (AI), and the swelling degree of paw edema were determined. After the secondary immunity, we detected the changes in body weight once every three days. The swelling degree of the paw was detected by a Vernier caliper. The specific method was as follows: a white point near the center of the foot plantar was selected as a reference, and the horizontal and vertical diameters were measured with the same width (width, W) and thickness (thickness T), respectively. The swelling of the paw was measured by paw area, and the formula of foot plantar area (area, A): A = PI × W × T/4. AI was tested every three to four days (0 = normal, 1 = mild swelling and erythema of digits or ankles, 2 = moderate swelling and erythema of digits or ankles, 3 = marked swelling of paws including digits, 4 = severe swelling and erythema with limited motion in many joints). This system yielded a total score between 0 and 16 for each mouse. The measurement process was performed by two independent double-blinded observers.

#### 3.4.3. Enzyme-Linked Immunosorbent Assay (ELISA)

On day 52, blood samples were collected from each mouse’s eye socket vein. Then, the sera were isolated by centrifugation at 10,000 rpm for 10 min after standing at room temperature for 20 min. Sera samples were determined by ELISA. According to the manufacturer’s instructions, processed samples’ optical density (OD) values were measured at 450 nm. There was a definite linear relationship between cytokine concentration and OD value, which was determined by the acknowledged standard curve sample concentrations of cytokines, after which the data were analyzed to sum up CRM influence on the inflammatory factors. All the data were presented as means ± standard deviation (SD).

### 3.5. Analysis of Spectrum-Effect Relationships

#### 3.5.1. Stepwise Multiple Linear Regression Analysis

Multivariate linear regression analysis was performed by using SPSS 16.0 software. The peak area of each peak (X1–X17) in the HPLC fingerprint was taken as the independent variable (X), and the content of each inflammatory factor was taken as the dependent variable (Y). The equations were established by the MLR stepwise to screening out peaks that had a significant contribution to the efficacy and anti-inflammatory effect.

#### 3.5.2. Partial Correlation Analysis

Using the Pearson correlation coefficient as the index, the common peaks in the different batches of CRM fingerprints were regarded as a set of variables, and the efficacy of the inflammatory indexes was observed as another group of variables. It was suggested that the correlation degree between peaks and efficacy indices can be predicted, including the correlation size, significant degree, and change direction.

#### 3.5.3. Gray Relational Analysis

We obtained the information sequences of the CRM anti-inflammatory effect using the four inflammatory indicators as proto-sequences and the quantitative fingerprint peak areas of different batches as subsequences, of which all data were processed by a data-processing system (DPS), and the correlation degrees of the subsequences were sorted by relation degree. Thus, the correlation degree of the sequences consisted of peaks that reflect their anti-inflammatory activity.

## 4. Conclusions

Our research found that there were similarities and differences in CRM fingerprints of different habitats and harvest years, and their anti-inflammation effects were not the same. In this study, we established the spectrum-effect relationships of HPLC-UV fingerprints and pharmacological experiments to determine the bioactive components related to the anti-inflammatory effect of CRM. The spectrum-effect relationships revealed that the alkaloids, saponins, and sapogenins together played a role in the compound’s anti-inflammatory efficacy. The results also showed that the correlation between saponins and inflammatory factors were significantly closer than that of alkaloids, and saponins linked with less sugar may have higher inhibition on inflammatory factors in CIA mice. This study laid a solid foundation for effective substances identification in CRM, and at the same time provided the basis for its quality control.

## Figures and Tables

**Table 1:** The relative retention time of 17 common peaks.

**Sample**	**Retention Time**								
	**X1**	**X2**	**X3**	**X4**	**X5**	**X6**	**X7**	**X8**	**X9**
**S1**	0.135	0.651	0.754	0.768	0.778	0.850	0.862	0.888	0.911
**S2**	0.134	0.650	0.753	0.767	0.777	0.849	0.862	0.888	0.911
**S3**	0.134	0.650	0.754	0.768	0.778	0.850	0.861	0.888	0.912
**S4**	0.133	0.649	0.752	0.766	0.777	0.848	0.860	0.888	0.911
**S5**	0.134	0.649	0.752	0.766	0.777	0.847	0.859	0.888	0.903
**S6**	0.134	0.648	0.752	0.766	0.777	0.848	0.860	0.888	0.911
**S7**	0.133	0.646	0.750	0.764	0.774	0.845	0.857	0.886	0.910
**S8**	0.132	0.645	0.749	0.763	0.774	0.845	0.859	0.885	0.909
**S9**	0.132	0.645	0.749	0.763	0.773	0.845	0.856	0.885	0.909
**S10**	0.132	0.645	0.749	0.763	0.774	0.845	0.856	0.885	0.909
**S11**	0.132	0.645	0.750	0.764	0.775	0.846	0.857	0.887	0.910
$\bar{x}$	0.133	0.647	0.751	0.765	0.776	0.847	0.795	0.887	0.910
**RSD%**	0.721	0.352	0.232	0.260	0.217	0.235	0.273	0.164	0.260
**Sample**	**Retention Time**								
	**X10**	**X11**	**X12**	**X13**	**X14**	**X15**	**X16**	**X17**	
**S1**	0.930	1.000	1.419	1.446	1.499	1.614	1.669	1.800	
**S2**	0.930	1.000	1.418	1.445	1.498	1.614	1.668	1.799	
**S3**	0.931	1.000	1.419	1.445	1.498	1.614	1.668	1.800	
**S4**	0.930	1.000	1.418	1.445	1.498	1.614	1.668	1.799	
**S5**	0.930	1.000	1.417	1.443	1.497	1.612	1.666	1.797	
**S6**	0.930	1.000	1.418	1.444	1.497	1.613	1.667	1.799	
**S7**	0.929	1.000	1.413	1.439	1.492	1.607	1.661	1.792	
**S8**	0.929	1.000	1.412	1.438	1.491	1.606	1.660	1.791	
**S9**	0.929	1.000	1.412	1.438	1.491	1.606	1.660	1.791	
**S10**	0.928	1.000	1.413	1.439	1.492	1.607	1.661	1.792	
**S11**	0.929	1.000	1.416	1.442	1.495	1.611	1.665	1.796	
$\bar{x}$	0.930	1.000	1.416	1.443	1.496	1.611	1.665	1.796	
**RSD%**	0.001	0.000	0.210	0.219	0.222	0.215	0.214	0.210	

**Table 2:** The relative peak area of 17 common peaks.

**Sample**	**Peak Area**								
	**X1**	**X2**	**X3**	**X4**	**X5**	**X6**	**X7**	**X8**	**X9**
**S1**	9.456	27.201	1.565	2.515	0.938	0.943	0.943	1.181	0.607
**S2**	7.074	21.749	1.319	2.144	0.900	1.346	0.767	1.346	0.702
**S3**	6.824	13.654	1.684	1.757	0.961	1.157	0.879	1.257	0.533
**S4**	8.613	19.969	1.899	2.195	0.974	0.906	0.948	1.236	0.769
**S5**	4.969	9.526	1.034	3.895	0.584	1.501	0.658	0.771	1.312
**S6**	10.942	21.792	1.645	1.931	0.922	0.600	0.599	1.083	0.553
**S7**	11.652	18.294	1.930	1.893	1.157	0.411	0.463	1.326	0.803
**S8**	12.413	18.635	1.626	1.874	0.881	0.374	0.756	1.487	0.781
**S9**	13.809	22.756	1.492	2.207	1.027	0.443	0.577	1.290	0.764
**S10**	15.058	25.958	2.264	2.615	1.262	0.977	0.959	1.725	0.629
**S11**	11.053	23.134	2.245	2.842	1.350	0.706	1.265	1.502	1.003
$\bar{x}$	10.157	20.040	1.695	2.373	1.002	0.842	0.763	1.296	0.910
**RSD%**	29.17	24.75	20.84	24.89	19.73	43.43	33.25	18.14	0.003
**Sample**	**Peak Area**								
	**X10**	**X11**	**X12**	**X13**	**X14**	**X15**	**X16**	**X17**	
**S1**	3.312	1.000	0.780	0.940	0.776	0.588	0.716	0.405	
**S2**	3.402	1.000	0.461	0.387	0.411	0.399	0.758	0.375	
**S3**	2.646	1.000	0.534	0.577	0.415	0.430	0.845	0.399	
**S4**	3.276	1.000	0.721	1.259	1.201	0.491	0.845	0.571	
**S5**	1.422	1.000	0.560	0.519	0.846	0.191	0.391	0.215	
**S6**	2.847	1.000	0.808	1.378	1.172	0.546	1.182	0.612	
**S7**	3.189	1.000	0.533	0.591	0.441	0.584	1.004	0.515	
**S8**	3.324	1.000	0.439	0.334	0.283	0.316	1.068	0.507	
**S9**	3.795	1.000	0.434	0.385	0.387	0.716	1.176	0.438	
**S10**	3.985	1.000	0.428	0.440	0.329	0.468	0.744	0.463	
**S11**	3.532	1.000	0.370	0.306	0.358	0.445	0.958	0.283	
$\bar{x}$	3.174	1.000	0.562	0.620	0.575	0.459	0.875	0.432	
**RSD%**	20.73	0.000	26.50	59.66	58.48	30.79	25.39	26.24	

**Table 3:** The fingerprint similarity evaluation data.

Sample	S1	S2	S3	S4	S5	S6	S7	S8	S9	S10	S11
**S1**	1.000	0.908	0.980	0.916	0.968	0.917	0.942	0.959	0.964	0.951	0.948
**S2**	0.908	1.000	0.915	0.862	0.904	0.891	0.902	0.905	0.903	0.909	0.891
**S3**	0.980	0.915	1.000	0.928	0.977	0.933	0.959	0.967	0.968	0.954	0.947
**S4**	0.916	0.862	0.928	1.000	0.920	0.906	0.919	0.921	0.916	0.908	0.908
**S5**	0.968	0.904	0.977	0.920	1.000	0.936	0.971	0.983	0.974	0.948	0.944
**S6**	0.917	0.891	0.933	0.906	0.936	1.000	0.953	0.952	0.946	0.942	0.941
**S7**	0.942	0.902	0.959	0.919	0.971	0.953	1.000	0.982	0.969	0.941	0.937
**S8**	0.959	0.905	0.967	0.921	0.983	0.952	0.982	1.000	0.980	0.957	0.948
**S9**	0.964	0.903	0.968	0.916	0.974	0.946	0.969	0.980	1.000	0.964	0.950
**S10**	0.951	0.909	0.954	0.908	0.948	0.942	0.941	0.957	0.964	1.000	0.956
**S11**	0.948	0.891	0.947	0.908	0.944	0.941	0.937	0.948	0.950	0.956	1.000
**R**	0.977	0.935	0.985	0.945	0.984	0.965	0.979	0.987	0.985	0.976	0.971

**Table 4:** Multiple comparison SNK test results of TNF-α(^a^: Alpha).

The Content of TNF-α								
	Group	N	Student for Alpha = 0.05					
			1	2	3	4	5	6
Student-Newman-Keuls ^a^	**Normal**	6	58.78					
	**S7**	6		73.80				
	**S8**	6		76.54				
	**S4**	6			78.18			
	**S6**	6			78.34			
	**S3**	6			78.93			
	**S10**	6				83.45		
	**S9**	6				83.91		
	**S5**	6				84.09		
	**S1**	6				84.42		
	**S2**	6					87.51	
	**S11**	6					88.19	
	**Vehicle**	6						118.67
	**Sig.**		1.00	1.00	0.107	0.783	0.510	1.00

**Table 5:** Multiple comparison SNK test results of IL-1β.

The Content of IL-1β										
	Group	N	Student for Alpha = 0.05							
			1	2	3	4	5	6	7	8
Student-Newman-Keuls ^a^	**Normal**	6	43.39							
	**S7**	6		50.32						
	**S8**	6			53.61					
	**S10**	6				60.11				
	**S9**	6					63.65			
	**S11**	6						82.40		
	**S4**	6						82.62		
	**S3**	6						83.32		
	**S6**	6						83.59		
	**S5**	6						83.99	83.99	
	**S1**	6							85.95	
	**S2**	6							85.99	
	**Vehicle**	6								120.49
	**Sig.**		1.000	1.000	1.000	1.000	1.000	0.395	0.072	1.00

**Table 6:** Multiple comparison SNK test results of IL-6.

The Content of IL-6										
	Group	N	Student for Alpha = 0.05							
			1	2	3	4	5	6	7	8
Student-Newman-Keuls ^a^	**Normal**	6	92.34							
	**S8**	6		103.01						
	**S7**	6		104.89	104.89					
	**S4**	6		106.37	106.37	106.37				
	**S3**	6		109.40	109.40	109.40	109.40			
	**S5**	6		112.13	112.13	112.13	112.13	112.13		
	**S10**	6		112.43	112.43	112.43	112.43	112.43		
	**S6**	6			113.97	113.97	113.97	113.97	113.97	
	**S9**	6				115.68	115.68	115.68	115.68	
	**S1**	6					117.42	117.42	117.42	
	**S2**	6						120.36	120.36	
	**S11**	6							122.37	
	**Vehicle**	6								152.93
	**Sig.**		1.000	0.055	0.071	0.060	0.148	0.130	0.085	1.00

**Table 7:** Multiple comparison SNK test results of IL-17.

The Content of IL-17								
	Group	N	Student for Alpha = 0.05					
			1	2	3	4	5	6
Student-Newman-Keuls ^a^	**Normal**	6	75.15					
	**S8**	6		86.52				
	**S7**	6		90.33	90.33			
	**S5**	6		91.75	91.75			
	**S6**	6		91.83	91.83			
	**S9**	6		92.93	92.93			
	**S10**	6		94.10	94.10	94.10		
	**S11**	6			97.64	97.64	97.64	
	**S1**	6			99.20	99.20	99.20	
	**S2**	6				102.14	102.14	
	**S3**	6					104.01	
	**S4**	6					105.63	
	**Vehicle**	6						129.14
	**Sig.**		1.000	0.165	0.084	0.059	0.092	1.00

**Table 8:** Integration of the analytical results.

Correlation Analysis	TNF-α	IL-1β	IL-6	IL-17
STEPWISE MLR	X2, X6	X1, X3, X6	X1, X13	X1, X2, X10, X15
PCA	X2, X11, X17	X1, X6, X7, X12, X13, X14	X2, X17	X1, X2, X7, X10, X15
in total	X2, X6, X11, X17	X1, X3, X6, X7, X12, X13, X14	X1, X2, X13, X17	X1, X2, X7, X10, X15

**Table 9:** The results of GRA between 17 characteristic peaks and sera cytokines.

	TNF-α		IL-1β		IL-6		IL-17	
	No.	Correlation	No.	Correlation	No.	Correlation	No.	Correlation
1	X17	0.7878	X17	0.7214	X8	0.9528	X17	0.9221
2	X15	0.7861	X15	0.7199	X5	0.9506	X15	0.9188
3	X12	0.7811	X12	0.7144	X11	0.9498	X12	0.9135
4	X14	0.7783	X14	0.7105	X7	0.9497	X14	0.9094
5	X13	0.7775	X13	0.7100	X3	0.9476	X13	0.9075
6	X9	0.7685	X9	0.7042	X16	0.9451	X9	0.8979
7	X7	0.7680	X16	0.7035	X6	0.9439	X7	0.8958
8	X16	0.7668	X7	0.7031	X13	0.9420	X16	0.8940
9	X6	0.7633	X5	0.6980	X12	0.9414	X6	0.8896
10	X5	0.7607	X6	0.6971	X14	0.9397	X5	0.8858
11	X11	0.7569	X11	0.6939	X9	0.9395	X11	0.8813
12	X8	0.7480	X8	0.6873	X4	0.9372	X8	0.8686
13	X3	0.7328	X3	0.6736	X15	0.9359	X3	0.8477
14	X4	0.7082	X4	0.6505	X17	0.9317	X4	0.8172
15	X10	0.6881	X10	0.6355	X10	0.9030	X10	0.7886
16	X1	0.5820	X1	0.5445	X1	0.7286	X1	0.6531
17	X2	0.5227	X2	0.4894	X2	0.6380	X2	0.5789

**Table 10:** Assignments of the correlated peaks.

No.	Mass Data		Compound	Aglycones	Formula	Mol. Wt.
X1	[M + H]^+^	205.1	*N*-methylcytisine	-	C_12_H_16_N_2_O	204
X2	[M + H]^+^	343.2	Magnoflorine	-	C_20_H_24_NO_4_	342
X3	[M + H]^+^	344.1	Unknown	-	-	343
X4	[M − H]^−^	1559.7	Leyemudanoside D	Caulophyllogenin	C_71_H_116_O_37_	1560
X5	[M − H]^−^	1251.6	Leyemudanoside C	Caulophyllogenin	C_59_H_96_O_28_	1252
X6	[M + Na]^+^	1111.5	Leyemudanoside G	11-oxo-Hederagenin	C_53_H_84_O_23_	1088
X7	[M − H]^−^	1251.6	Leyemudanoside B	Caulophyllogenin	C_59_H_96_O_28_	1252
X8	[M − H]^−^	1251.2	Cauloside H	Caulophyllogenin	C_59_H_96_O_28_	1252
X9	[M − H]^−^ [M + HCOOH − H]^−^	1089.0 1135.1	Leonticin D	Caulophyllogenin	C_53_H_86_O_23_	1090
X10	[M − H]^−^	1235.2	Cauloside G	Hederagenin	C_59_H_96_O_27_	1236
X11	[M − H]^−^ [M + HCOOH − H]^−^	1073.0 1119.1	Cauloside D	Hederagenin	C_53_H_86_O_22_	1074
X12	[M − H]^−^ [2M − H]^−^	618.91 1239.5	Cauloside B	Caulophyllogenin	C_35_H_56_O_9_	620
X13	[M − H]^−^ [M + Na]^+^	765.07 789.08	Cauloside C	Hederagenin	C_41_H_66_O_13_	766
X14	[M − H]^−^	603.3	Cauloside A	Hederagenin	C_35_H_56_O_8_	604
X15	[M − H]^−^	455.3	Oleanic acid	-	C_30_H_48_O_3_	456
X16	[M − CH_3_]^−^	399	β-sitosterol	-	C_29_H_50_O	414
X17	[M − H]^−^	749.1	Unknown	-	-	750

**Table 11:** The CRM samples with their different harvest times and habitats.

Sample No.	Collection Time	Origin
**S1**	September 2013	Dazhou mountain in Sichuan Province, China
**S2**	September 2013	Dazhou mountain in Sichuan Province, China
**S3**	September 2014	Mao county in Sichuan Province, China
**S4**	August 2014	Mao county in Sichuan Province, China
**S5**	September 2014	Anguo city in Hebei Province, China
**S6**	September 2014	Anguo city in Hebei Province, China
**S7**	September 2014	Suiling Zhangjiawan forest farm in Heilongjiang Province, China
**S8**	September 2014	Suiling Zhangjiawan forest farm in Heilongjiang Province, China
**S9**	September 2013	Suiling Zhangjiawan forest farm in Heilongjiang Province, China
**S10**	September 2013	Suiling Zhangjiawan forest farm in Heilongjiang Province, China
**S11**	September 2012	Suiling Zhangjiawan forest farm in Heilongjiang Province, China

## References

[1] Lau C.S., Gibofsky A., Damjanov N., Lula S., Marshall L., Jones H., Emery P. (2017). Down-titration of biologics for the treatment of rheumatoid arthritis: A systematic literature review. Rheumatol. Int..

[2] Lü S.W., Wang Q.S., Li G.Y., Sun S., Guo Y.Y., Kuang H.X. (2015). The treatment of rheumatoid arthritis using Chinese medicinal plants: From pharmacology to potential molecular mechanisms. J. Ethnopharmacol..

[3] Choy E., Panayi G. (2001). Cytokine pathways and joint inflammation in rheumatoid arthritis. N. Engl. J. Med..

[4] Ma Y.M., Xing H., Liu J.J., Kang H.X. (2012). The study of Chemical composition from *Caulophyllum robustum* Maxim which belong to Taibai seven medicine. J. Anhui Agric. Sci..

[5] Campbell I.K., Hamilton J.A., Wicks I.P. (2000). Collagen-induced arthritis in C57BL/6 (H-2b) mice: New insights into an important disease model of rheumatoid arthritis. Eur. J. Immunol..

[6] Song H., Qiao F., Atkinson C., Holers V.M., TomLinson S. (2007). Acomplement C3 inhibitor specially targeted to sites of complement activation effectively ameliorates collagen-induced arthritis in DBA/1 J mice. J. Immunol..

[7] Holmdahl R., Bockermann R., Bäcklund J., Yamada H. (2002). The molecular pathogenesis of collagen-induced arthritis in mice-a model for rheumatoid arthritis. Ageing Res. Rev..

[8] Song L.R., Hong X., Ding X.L., Zang Z.Y. (2001). Modern Zhong Yao Da Ci Dian: Volume One.

[9] Xia Y.G., Li G.Y., Liang J., Yang B.Y., Lü S.W., Kuang H.X. (2014). Genus Caulophyllum: An Overview of Chemistry and Bioactivity. Evid.-Based Complement. Altern. Med..

[10] Lee Y., Jung J.C., Ali Z., Khan I.A., Oh S. (2012). Anti-Inflammatory Effect of Triterpene Saponins Isolated from Blue Cohosh (*Caulophyllum thalictroides*). Évid.-Based Complement. Altern. Med..

[11] Yang X.F., Ma Y.M., Xing H., Liu J.J., Kang Y.X. (2013). Studies on triterpene saponins and their biological activity of *Caulophyllum robustum*. J. Shaannxi Univ. Sci. Technol..

[12] Lü S.W., Su H., Yu F.M., Guo Y.Y., Kuang H.X. (2017). Therapeutic Effect and Mechanism of *Caulophyllum robustum* Maxim Extract on Adjuvant Arthritis Rats. Tradit. Chin. Drug Res. Clin. Pharmacol..

[13] Xiao L.W., Bing R.L., Chien K.C., Jun R.W., Shoei S.L. (2011). Four new fluorenone alkaloids and one new dihydroazafluoranthene alkaloid from *Caulophyllum robustum* Maxim. Fitoterapia.

[14] Qin K.M., Zheng L.J., Shen B.J., Zhang X.H., Li H., Di L.Q., Xu Z.S., Cai C. (2013). Application of spectrum-effect relationship in Chinese medicine research and related thinking. China J. Chin. Mater. Med..

[15] Huo S.X., Kang Y.T., Peng X.M., Gao L., Yan M. (2013). Spectrum-effect relationship of extract from rhizome of *Alpinia officinarum* on promotion of melanogenesis. Chin. Tradit. Herb. Drugs.

[16] Liu N., Li J., Li B.G. (2014). Application of multivariate statistical analysis and thinking in quality control of Chinese medicine. China J. Chin. Mater. Med..

[17] Shen Y.W., Liu J.J. (2010). Application of the Gray Relevancy Method to College Teachers’ Research Performance Assessment of Humanities and Social Sciences. Sci. Technol. Manag. Res..

[18] Lü S.W., Dong S.Y., Guo Y.Y., Sun S., Kuang H.X. (2015). Advance in Application of Data Analysis Technique in Spectrum-effect Relationship of Traditional Chinese Medicines. Chin. J. Exp. Tradit. Méd. Formulae.

[19] Han Y.P., Lei Z.H., Zhao D. (2015). Influence Factors and Countermeasures of Quality of Traditional Chinese Medicine. Mod. Agric. Sci. Technol..

[20] Sun M.Y., Wu W.J., Xu H.L., Liu X., Xia S., Shao Q.X. (2012). Methotrexate inhibited the development of chllagen-induced arthritis in mice by decreasing the ratio of Th17/Treg cells. J. Jiangsu Univ. (Med. Ed.).

[21] Zheng H.M., Jin S. (2013). Effects of Glucosidorum Tripterygll Totorum Tablet on Serum HMGB1 and IL-17 in Rats with Collagen-induced Arthritis. Chin. J. Exp. Tradit. Med. Formulae.

[22] Nagy G., Clark J.M., Buzas E., Gorman C., Pasztoi M., Koncz A., Falus A., Cope A.P. (2008). Nitric oxide production of T lymphocytes is increased in rheumatoid arthritis. Immunol. Lett..

[23] Yang C.L., Or T.C., Ho M.H., Lan A.S. (2013). Scientific Basis of Botanical Medicine as Alternative Remedies for Rheumatoid Arthritis. Clin. Rev. Allergy Immunol..

[24] Kugyelka R., Kohl Z., Olasz K., Ranch T.A., Glant T.T., Boldizsar F. (2016). Enigma of IL-17 and Th17 Cells in Rheumatoid Arthritis and in Autoimmune Animal Models of Arthritis. Mediat. Inflamm..

[25] Mclnnes I.B., Schett G. (2007). Cytokines in the pathogenesis of rheumatoid arthritis. Nat. Rev. Immunol..

[26] Li G.Y. (2006). Studies on Chemical Constituents of Active Fraction from *Caulophyllum robustum* for Anti-Rheumatism. Master’s Thesis.

[27] Xia Y.G., Li G.Y., Liang J., Ortori C.A., Yang B.Y., Kuang H.X., Barrett D.A. (2014). A strategy for characterization of triterpene saponins in *Caulophyllum robustum* hairy roots by liquid chromatography with electrospray ionization quadrupole time-of-flight mass spectrometry. J. Pharm. Biomed. Anal..

[28] Lü S.W., Li G.Y., Zuo Y.M., Kang H.X. (2005). Determination the content of total saponins from *Caulophyllum robustum* Maxim. Acta Tradit. Chin. Med..

[29] Li G.Y., Xu N., Lü S.W., Kang H.X. (2015). Saponin constituents from roots and rhizomes of *Caulophyllum robustum*. Chin. Tradit. Herb. Drugs.

[30] Aminin D.L., Agafonova I.G., Gnedoi S.N., Strigina L.I., Anisimov M.M. (1999). The effect of pH on biological activity of plant cytotoxin Cauloside C. Comp. Biochem. Physiol. A Mol. Integr. Physiol..

[31] Anisimov M.M., Shentsova E.B., Shcheglov V.V., Shulmilov Y.N., Rasskazov V.A., Striqina L.I., Chetyrina N.S., Elyakov G.B. (1978). Mechanism of cytotoxic action of some triterpene glycosides. Toxicon.

[32] Küpeli E., Kosar M., Yesilada E., Hüsnü K., Baser C. (2002). A comparative study on the anti-inflammatory, antinociceptive and antipyretic effects of isoquinoline alkaloids from the roots of Turkish Berberis species. Life Sci..

[33] Yamashita R., Fujiwara Y., Ikari K., Hamada K., Otomo A., Yasuda K., Noda M., Kaburagi Y. (2007). Extracellular proteome of human hepatoma cell, HepG2 analyzed using two-dimensional liquid chromatography coupled with tandem mass spectrometry. Mol. Cell. Biochem..

[34] Sheheglov V.V., Anisimov M.M., PoPov A.M., Kiseleva M.I., Sebko I.G. (1979). Effect of trierpene glyeosides on plasma membrane permeability for UV-absorbing substances in *Saccharomyces carlsbergensis* yeast cells. Antibiotiki.

[35] Anisimov M.M., Ivanova A.S., Popov A.M., Kiseleva M.I., Sebko I.G. (1981). Effect of trierpene glyeosides and polyene antibiotics on cell membrane permeability for K^+^ ions and UV-absorbing substances. Prikl. Biokhim. Mikrobiol..

[36] Anisimov M.M., Shcheglov V.V., Kiseleva M.I. (1978). Effect of trierpene glyeosides on the plasma membrane permeability for amino acids in *Saccharomyces carlsbergensis* yeast cells. Antibiltiki.

[37] Tian L.T., Ma L., Zhe N.S. (2002). Survey of Pharmacology of Aleanolic Acid. China J. Chin. Mater. Med..

[38] Kapi1 A., Sharma S. (1995). Effect of Oleanolic Acid on completment in Adjuvant and Carrageenan-induced Inflammation in Rats. J. Pharm. Pharmacol..

[39] Kim Y.P., Lee E.B., Kim S.Y., Li D., Ban H.S., Lim S.S., Shin K.H., Ohuchi K. (2001). Inhibition of rostaglandin E2 production by platycodin D isolated from the root of *Platycodon gradiflorum*. Planta Med..

[40] Lee C.W., Park S.M., Zhao R., Lee C., Chun W., Son Y., Kim S.H., Jung J.Y., Jegal K.H., Cho I.J. (2015). Hederagenin, a major component of *Clematis mandshurica* Ruprecht root, attenuates inflammatory responses in RAW 264.7 cells and in mice. Int. Immunopharmacol..

[41] Guo T. (2011). Studies on the Chemical Constituents of *Zanthoxylum armatum* DC. and *Z. avicenna* (Lam.) DC. and Analgesic, Anti-inflammatory Activities of *Z. armatum*. Master’s Thesis.

[42] Tsypysheva I.P., Borisevich S.S., Zainullina L.F., Makara N.S., Koval’skaya A.V., Petrova P.R., Khursan S.L., Vakhitova Y.V., Zarudii F.S. (2017). Anti-Inflammatory Activity of Novel 12-*N*-methylcytisine Derivatives. Anti-Allergy Agents Med. Chem..

[43] Xiao Z.B., Liu X.L., Cheng R.Q., Jia H.X., Wang X.Y., Kong L.N., Li Y.J., Cui T.T., Qu H.D., Feng C. (2016). Influence of β-sitosterol on Gastric Mucosal Side Effect Induced by Aspirin and Its Pharmacological Functions. Chin. J. Exp. Tradit. Med. Formulae.

[44] Zhang S.J., Chen Q.B. (2003). The application of statistical analysis software SPSS (V), correlation analysis and regression analysis. Anim. Husb. Vet. Med..

[45] Wang M.Z., Jing S.L., He X.J., Lv C., Li J., Luo D., Lv A.P. (2013). The association studies about CIA rats foot plantar size and joint. Chin. J. Basic Med. Tradit. Chin. Med..

